# Assessing sleep quality in elite and junior cyclists

**DOI:** 10.3389/fspor.2024.1369435

**Published:** 2024-04-30

**Authors:** Alejandro Javaloyes, Manuel Mateo-March, Iván Peña-González, Manuel Moya-Ramón

**Affiliations:** Department of Sport Science, Sports Research Centre, Miguel Hernández University of Elche, Elche, Spain

**Keywords:** sleep efficiency, athlete performance, recovery, elite sports, endurance

## Abstract

In the pursuit of optimal recovery, the significance of sleep cannot be overstated for elite cyclists, including high-level cyclists within the junior category. This study aims to assess the sleep quality of elite athletes of different categories and disciplines, including junior. The sleep quality of 112 high-level cyclists (males *n* = 80; females *n* = 32) participating in endurance and sprint disciplines was evaluated using the Pittsburgh Sleep Quality Index (PSQI). A noteworthy 41% of both elite and junior cyclists displayed poor sleep quality. No significant differences were observed between elite and junior cyclists in terms of sleep quality, but there was a medium effect size, indicating greater sleep efficiency in junior cyclists [0.36 (0.16, 0.53)]. Gender differences were found, with females exhibiting worse PSQI scores (males = 4.00 [2.25]; females 5.00 [3.00]; *p* = 0.035). Endurance cyclists spent more time in bed compared to cyclists from sprinting disciplines (8:30 [1.00] and 8:00 [1:03], respectively; *p* = 0.019). These findings reveal poor sleep habits, even among individuals classified as good sleepers by the PSQI, emphasizing the importance of preventing sleep disorders in cyclists. This study provides valuable insights into athlete sleep quality, encompassing various categories, genders, and cycling disciplines. In conclusion, elite cyclists demonstrate suboptimal sleep quality, suggesting the potential for interventions utilizing the PSQI. These findings advocate for the incorporation of sleep quality assessments into routine evaluations for athletes.

## Introduction

For elite athletes who perform at a high level, sleep is of paramount importance for optimal health and performance. While numerous studies have documented the effects of sleep deprivation in the general population (1), limited research exists specifically examining its effects on high-level athletes (2). However, emerging evidence suggests that elite athletes are particularly susceptible to habitual short sleep duration, typically getting less than seven hours of sleep each night (3). Sleep deprivation can negatively affect various aspects of athletic performance (4, 5), including reaction time, accuracy, strength endurance, and cognitive function (6, 7). Conversely, increasing both the quantity and quality of sleep has been associated with improved performance in areas directly related to the demands of the sport, such as reaction times, coordination, split-second decision-making, and glucose metabolism (8). Sleep disturbances in athletes can occur at two distinct time points: prior to important competitions and during normal training. Poor sleep habits, such as the exposition to blue light devices such as watching television in bed or smartphones, nocturnal bathroom visits, caffeine consumption, and excessive mental activity before bedtime, can disrupt sleep during regular training. Therefore, it is crucial for athletes to consider their sleep habits, optimal sleep timing, and duration to achieve peak performance.

Cycling, as a demanding endurance sport, places substantial physical and psychological stress on athletes. Elite cyclists invest considerable time and effort in training, competition, and recovery, potentially disrupting their sleep patterns (9, 10). Endurance cycling involves prolonged exertion and may entail multi-day races or intense training blocks, presenting unique challenges for maintaining optimal sleep. Conversely, sprint cycling disciplines, such as track cycling, typically involve shorter, high-intensity efforts, potentially affecting sleep quality differently (11).

While previous research has explored sleep-related aspects in athletes from various disciplines, such as team sports or track and field, the unique demands and characteristics of cycling warrant an independent examination of sleep quality in this specific population. The extensive time and effort dedicated to training, competing, and recovering in elite cyclists can significantly impact their sleep patterns. Furthermore, endurance cycling's prolonged exertion as in ultra-endurance races and events can pose additional challenges in maintaining adequate sleep duration and quality (12). Understanding the specific sleep challenges faced by elite cyclists is crucial for developing targeted interventions and strategies to promote adequate sleep and ultimately improve performance and well-being.

Analysing the sleep quality of younger cyclists is of paramount importance for several reasons. Firstly, this age group represents the developmental stage where athletes transition from junior to elite levels, experiencing significant physical and psychological changes. Sleep plays a crucial role in supporting these physiological adaptations and facilitating optimal growth, recovery, and performance. Previous research has supported several dysfunctions in sleep quality in young athletes (13). Therefore, understanding the sleep patterns and quality of junior cyclists can provide valuable insights into their specific sleep needs and challenges, allowing for targeted interventions to optimize their sleep habits and promote healthy development. Additionally, investigating sleep in this age group helps identify potential issues early on, enabling the implementation of effective strategies to establish healthy sleep routines that can benefit their long-term athletic careers. Furthermore, examining sleep quality in junior cyclists contributes to a comprehensive understanding of sleep-related factors affecting performance across different stages of an athlete's career. This knowledge can inform coaches, trainers, and sports scientists in developing evidence-based training programs and support systems that prioritize sleep as a critical component of overall well-being and success in young cyclists.

This study seeks to bridge the knowledge gap regarding sleep quality in high-level cyclists by comprehensively evaluating the sleep patterns and quality of junior and elite athletes participating in endurance and sprint cycling disciplines. In examining the sleep characteristics of this population, this study aims to identify potential sleep challenges that may impact their performance. The hypothesis of the study is that high-level cyclists will display poor sleep quality, irrespective of their categories. Additionally, female cyclists are expected to exhibit worse sleep quality than their male counterparts.

## Method

### Participants and study design

A total of 112 cyclists (males *n* = 80; females *n* = 32) participate in this study. The cyclists were members of the national team of the Spanish cycling federation teams in junior (*n* = 60), U23 (*n* = 32) and elite (*n* = 20) categories. For this study, cyclists were categorized either as endurance cyclists [efforts >60 s; those belonging to road, mountain bike, or long-duration track disciplines (*n* = 64)] or sprint cyclists [efforts <60 s; sprint track cyclists or BMX riders (*n* = 48)]. This study was conducted in strict accordance with the principles outlined in the Helsinki Declaration. All procedures and protocols were reviewed and approved by the Universidad Miguel Hernández de Elche (Ref: DPS.JSM.02.18), and written informed consent was obtained from all participants prior to their involvement in the study.

Participants were recruited during a training camp placed at the beginning of the season, just after their pre-season. The training camp was placed after the first six weeks of training after the off-season period, during the month of December. Food, hydration status, and stimulant consumption, such as caffeine, were controlled and cyclists were asked to maintain their daily habits. During this period, cyclists typically followed their regular training regime plus different evaluations to assess their health and performance level. These include regular medical screening, body composition assessment and specific maximal performance tests. As a part of the training camp, sleep quality was assessed using the Pittsburgh Sleep Quality Index (PSQI) (14, 15). The PSQI was administered in the first day of the training camp.

### Questionnaire

The Pittsburgh Sleep Quality Index (PSQI) is a self-report questionnaire in which participants reported their typical sleep patterns over the preceding month. PSQI comprises 19 individual items organized into seven key components aimed at assessing diverse facets of sleep quality and disturbances in adults. These components encompass subjective sleep quality, sleep latency, sleep duration, habitual sleep efficiency, sleep disturbances (including nighttime awakenings, bathroom visits, and discomfort), use of sleep medications, and daytime dysfunction (evaluating issues like daytime fatigue and reduced productivity). Each component is scored based on participant responses, typically ranging from 0 to 3 points for each item, with higher scores indicating poorer sleep quality in most cases, except for sleep duration, where a longer duration corresponds to a lower score. The summation of individual component scores yields a global PSQI score, ranging from 0 to 21, which serves as an indicator of overall sleep quality. A global PSQI score exceeding 5 suggests poor sleep quality, while a score of 5 or lower generally indicates good sleep quality (14).

### Statistical analysis

All analyses were conducted using JASP (16). Before performing statistical analyses, the normality of all data was assessed both visually through Q-Q plots and objectively using the Shapiro-Wilk test. All variables showed significant deviations from a normal distribution (*p* < 0.05); therefore, non-parametric tests were employed. To compare differences between the groups, the Mann-Whitney test was utilized. Measures are presented as median and interquartile range (IQR) due to the ordinal nature of the data. Statistical significance was set at *p* < 0.05. Effect sizes (r for ordinal and continuous data) along with 95% confidence intervals (CI) were computed to evaluate the magnitude of differences (17). Effect sizes were interpreted as follows: trivial ≤0.10, small ≤0.3, medium ≤0.5, and large >0.5.

## Results

### General and categories

For all the cyclists (*n* = 112), the time in bed, sleep efficiency and sleep onset latency had median values of 8 h:17 min (1 h:19 min), 94.12% (9.10%) and 0 h:49 min (1 h:00 min), respectively. The PSQI median and IQR [median (IQR)] for sleep quality, sleep latency, sleep duration, sleep efficiency, sleep disturbances, sleep medication and daytime dysfunction were 1.00 (1.00), 1.00 (1.00), 0.00 (1.00), 0.00 (0.00), 0.00 (0.00), 0.00 (0.00), 0.00 (1.00), respectively. The median score for PSQI was 4.00 (3.00), with 41% of cyclist scoring >=5.

When cyclists were divided/clustered by type of effort, no statistical differences (*p* > 0.05) were obtained in any variables for endurance vs. sprint disciplines (Table 1). However, time in bed was slightly greater in endurance cyclists [8.5 (0.96) vs. 8.0 (0.89); ES = 0.26 (0.05, 0.45; small)].

**Table 1:** Descriptive data for characteristics, Pittsburgh sleep quality Index (PSQI) components, and total score for endurance and sprint cycling disciplines.

	Endurance (*n* = 64)	Sprint (*n* = 48)	*p*	ES (95% CI)
Characteristics				
Time in bed (h:min)	8:30 (1.00)	8:00 (1:03)	0.019	0.26* (0.05, 0.45) small
Sleep efficiency (%)	94.12 (8.61)	94.12 (10.34)	0.402	−0.09 (−0.30, 0.12); trivial
Sleep onset latency (h:min)	01:02 (01:01)	00:42 (00:59)	0.149	0.16 (−0.06, 0.36); trivial
PSQI components (0–3 score; Arbitrary Units, AU)				
Sleep quality	1.00 (1.00)	1.00 (1.00)	0.108	−0.01 (−0.30, 0.12); trivial
Sleep latency	1.00 (1.00)	2.00 (1.00)	0.744	−0.03 (−0.25, 0.18); trivial
Sleep duration	0.00 (1.00)	0.00 (1.00)	0.543	−0.06 (−0.27, 0.16); trivial
Sleep efficiency	0.00 (0.00)	0.00 (0.00)	0.740	0.02 (−0.19, 0.24); trivial
Sleep disturbances	1.00 (0.00)	1.00 (0.00)	0.532	−0.05 (−0.26, 0.17); trivial
Sleep medication	0.00 (0.00)	0.00 (0.00)	0.876	0.00 (−0.21, 0.22); trivial
Daytime dysfunction	0.00 (1.00)	1.00 (1.25)	0.089	−0.17 (−0.37, 0.04); trivial
PSQI Total				
Total (AU)	4.00 (2.00)	5:00 (3.00)	0.093	−0.18 (−0.38, 0.03); trivial
Proportion PSQI ≥5 (%)	35%	46%		

Data are presented as the median and interquartile range; categorical data are presented as a percentage. PSQI Total, overall sleep quality score from the PSQI; Proportion ≥5, the percentage of athletes scoring 5 AU or above on the PSQI, Effect sizes interpreted as trivial, ≤0.1; small, ≤0.3; medium ≤0.5; and large, >0.5, AU, arbitrary units; CI, confidence interval; PSQI, Pittsburgh Sleep Quality Index.

* *p* < 0.01.

Additionally, we found a similar lack of differences between male and female participants (Table 2). Males comprised 38.4% (*n* = 43) of the junior category and 33.0% (*n* = 37) of the elite category, while females accounted for 15.2% (*n* = 17) of juniors and 13.4% (*n* = 15) of elites. In terms of sleep quality, 66.3% (*n* = 53) of male participants were classified as good sleepers, contrasted with 33.8% (*n* = 27) as bad sleepers. For females, 40.6% (*n* = 13) were deemed good sleepers, with a proportion of 59.4% (*n* = 19) categorized as bad sleepers. Moreover, the division into endurance and sprint disciplines revealed equal participation among males, with each discipline comprising 50% (*n* = 40) of the male cohort. Female participation was skewed towards endurance, with 75% (*n* = 24) engaging in endurance disciplines compared to 25% (*n* = 8) in sprint.

**Table 2:** Descriptive data for characteristics, Pittsburgh sleep quality Index (PSQI) components, and total score for male and female.

	Male (*n* = 80)	Female (*n* = 32)	*p*	ES (95% CI)
Characteristics				
Time in bed (h:min)	8:28 (1.02)	8.07 (1.26)	0.643	0.06 (−0.18, 0.29); trivial
Sleep efficiency (%)	94.12 (11.11)	94.12 (10.58)	0.283	0.13 (0.11, 0.35); trivial
Sleep onset latency (h:min)	00:45 (01:02)	01:10 (00:56)	0.244	−0.14 (−0.36, 0.10); trivial
PSQI components (0–3 score; Arbitrary Units, AU)				
Sleep quality	1.00 (1.00)	1.00 (1.00)	0.089	−0.18 (−0.40, 0.05); trivial
Sleep latency	1.00 (1.00)	2.00 (1.00)	0.202	−0.14 (−0.37, 0.09); trivial
Sleep duration	0.00 (1.00)	0.00 (1.00)	0.116	−0.16 (−0.38, 0.08); trivial
Sleep efficiency	0.00 (0.00)	0.00 (0.25)	0.108	−0.13 (−0.35, 0.11); trivial
Sleep disturbances	1.00 (0.00)	1.00 (0.00)	0.129	−0.13 (−0.36, 0.10); trivial
Sleep medication	0.00 (0.00)	0.00 (0.00)	0.565	−0.03 (−0.26, 0.21); trivial
Daytime dysfunction	0.00 (1.00)	0.50 (1.00)	0.635	−0.05 (−0.28, 0.18); trivial
PSQI Total				
Total (AU)	4.00 (2.25)	5:00 (3.00)	0.035	−0.25 (−0.46, −0.02); small
Proportion PSQI ≥5 (%)	33%	59%		

Data are presented as the median and interquartile range; categorical data are presented as a percentage. PSQI Total, overall sleep quality score from the PSQI; Proportion ≥5, the percentage of athletes scoring 5 AU or above on the PSQI. Effect sizes interpreted as trivial, ≤0.1; small, ≤0.3; medium ≤0.5; and large, >0.5. AU, arbitrary units; CI confidence interval; PSQI, Pittsburgh Sleep Quality Index.

Finally, those cyclists reported as bad sleepers (PSQI Index >5; 41% of all the participants) scored higher in all the PSQI components apart from sleep medication (Table 3).

**Table 3:** Descriptive data for characteristics, Pittsburgh sleep quality Index (PSQI) components, and total score for bad and good sleepers, assessed by the PSQI.

	Good sleepers (*n* = 66)	Bad sleepers (*n* = 46)	*p*	ES (95% CI)
Characteristics				
Time in bed (h:min)	8:28 (1:02)	8:07 (1.26)	<.001	0.42 (0.23, 0.58); moderate
Sleep efficiency (%)	96.42 (8.66)	91.15 (7.23)	0.024	0.25 (0.04, 0.44); small
Sleep onset latency (h:min)	00:45 (01:02)	01:10 (00:56)	0.013	0.28 (0.07, 0.46); trivial
PSQI components (0–3 score; Arbitrary Units, AU)				
Sleep quality	0.00 (1.00)	1.00 (0.00)	<.001	−0.59 (−0.72, −0.43); large
Sleep latency	1.00 (1.00)	2.00 (1.00)	<.001	−0.37 (−0.54, −0.17); medium
Sleep duration	0.00 (0.00)	1.00 (1.00)	<.001	−0.59 (−0.72, −0.43); large
Sleep efficiency	0.00 (0.00)	0.00 (1.00)	<.001	−0.28 (−0.47, −0.07); small
Sleep disturbances	1.00 (1.00)	1.00 (0.00)	<.001	−0.38 (−0.55, −0.18); medium
Sleep medication	0.00 (0.00)	0.00 (0.00)		
Daytime dysfunction	0.00 (1.00)	1.00 (2.00)	<.001	−0.50 (−0.65, 0.32); large
PSQI Total				
Total (AU)	3.00 (2.00)	6:00 (2.00)	<.001	−0.99 (−1.00,−0.99); large

Data are presented as the median and interquartile range; categorical data are presented as a percentage. PSQI Total, overall sleep quality score from the PSQI; Proportion ≥5, the percentage of athletes scoring 5 AU or above on the PSQI. Effect sizes interpreted as trivial, ≤0.1; small, ≤0.3; medium ≤0.5; and large, >0.5. AU arbitrary units; CI, confidence interval; PSQI, Pittsburgh Sleep Quality Index.

## Discussion

Sleep quality plays a pivotal role in athletes’ performance and overall well-being. In this study, we investigated various aspects of sleep quality concerning different categories of cyclists, gender, and the type of effort they engage in. Moreover, we examined the specific components of the Pittsburgh Sleep Quality Index (PSQI) that might exert a greater influence on overall poor sleep quality scores. The main result of the present study is that a high proportion of elite and junior cyclists showed a dysfunctional sleep quality (41%). Furthermore, among those deemed as good sleepers (PSQI <5), different variables showed negative values that may compromise rest and promote bad sleep quality in the future. This fact highlights the need for educational interventions in high-level athletes, even if they show good sleep quality. The PSQI index may be implemented as a “pre-diagnosed” tool to detect the specific sleep disruptors that may lead to individualizing the educational process. Previous results reported even higher percentages (50.8%) in high-level athletes of individual sports (3). One possible explanation for the lower percentage of bad sleepers in our study could be that our intervention was placed before the competitive period. In this line, there is a correlation between heightened cognitive arousal during night-time and objective sleep disturbances (18) and this could happen during competition phases in this population (19). In contrast, another study in cross-country riders found better sleep quality (≈33% of bad sleepers) but similar time in bed and sleep efficiency that our study. However, the sample size of the mentioned study was limited to only thirteen cyclists, and the results must be taken with caution (20).

Our findings suggest that, with the exception of a enhanced sleep efficiency observed in junior cyclists and a slight, albeit statistically non-significant, increase in time spent in bed among elite cyclists, there are no substantial disparities in the majority of sleep quality parameters between these two athlete cohorts. This suggests that experience and training level may not directly be related to sleep quality in this population. These findings align with some prior research demonstrating that the level of competition does not consistently correlate with sleep quality in athletes (21). However, it is crucial to note that other studies have reported an inverse relationship between competition level and sleep quality (22), implying that this relationship may hinge on various factors, including specific competitive demands and sleep management strategies. As mentioned, elite cyclists exhibited lower sleep efficiency values (Table 4). It could be postulated that elite cyclists recognize the significance of sleep in their recovery but may not achieve the same level of efficiency in their sleep patterns as junior cyclists. It is noteworthy that sleep duration tends to diminish over one's lifespan (8). Consequently, this finding underscores the imperative of implementing sleep education strategies within this population, extending beyond the scope of young cyclists alone.

**Table 4:** Descriptive data for characteristics, Pittsburgh sleep quality Index (PSQI) components, and total score for junior and elite cyclists.

	Junior (*n* = 60)	Elite (*n* = 52)	*p*	ES (95% CI)
Characteristics				
Age (years)	17.02 (0.75)	23.50 (2.73)	0.001	−3.34* (−3.92, −2.762); *large*
Time in bed (h:min)	8.08 (1.13)	8.50 (1.25)	0.059	−0.21 (−0.40, 0.01); *small*
Sleep efficiency (%)	96.42 (8.66)	91.15 (7.23)	0.001	0.36* (0.16, 0.53); *medium*
Sleep onset latency (h:min)	00:49 (01:01)	00:49 (00:55)	0.357	0.10 (−0.11, 0.31); *trivial*
PSQI components (0–3 score; Arbitrary Units, AU)				
Sleep quality	1.00 (1.00)	1.00 (1.00)	0.909	0.12 (−0.20, 0.22); *trivial*
Sleep latency	1.00 (1.00)	2.00 (1.00)	0.430	0.00 (−0.21, 0.21); *trivial*
Sleep duration	0.00 (1.00)	0.00 (1.00)	0.938	−0.01 (−0.22, 0.20); *trivial*
Sleep efficiency	0.00 (0.00)	0.00 (0.00)	0.178	−0.10 (−0.30, 0.12); *trivial*
Sleep disturbances	1.00 (0.00)	1.00 (0.00)	0.717	0.03 (−0.18, 0.24); *trivial*
Sleep medication	0.00 (0.00)	0.00 (0.00)	0.240	0.05 (−0.17, 0.26); *trivial*
Daytime dysfunction	0.50 (1.00)	0.00 (1.00)	0.911	−0.01 (−0.22, 0.20); *trivial*
PSQI Total				
Total (AU)	4.00 (2.25)	4:00 (3.00)	0.637	−0.05 (−0.26, 0.16); *trivial*
Proportion PSQI ≥5 (%)	36%	46%		

Data are presented as the median and interquartile range; categorical data are presented as a percentage. PSQI Total, overall sleep quality score from the PSQI; Proportion ≥5, the percentage of athletes scoring 5 AU or above on the PSQI. Effect sizes interpreted as trivial, ≤0.1; small, ≤0.3; medium ≤0.5; and large, >0.5. AU, arbitrary units; CI, confidence interval; PSQI, Pittsburgh Sleep Quality Index.

* *p* < 0.01.

Regarding the differences between endurance and sprint cycling disciplines, while almost all sleep quality measures did not reveal significant differences between endurance and sprint cyclists, a significant discrepancy emerged in the PSQI total score, with the cyclists from sprint disciplines exhibiting higher scores indicative of poorer sleep quality. In addition, time in bed was greater in the endurance cyclist. These results align with previous observations in high-level athletes reporting small, better sleep quality in endurance athletes compared to strength and speed athletes (3). It is important to consider that differences in sleep quality may be linked to the specific training and competition demands in each discipline, as well as the sleep management strategies adopted by athletes.

In our study, we observed significant differences in sleep quality between males and females, with significantly higher PSQI total scores in females [Table 2; *p *= 0.035; −0.25 (−0.46, −0.02)]. In addition, 59% of the female participants were deemed as bad sleepers while men exhibited 33% of bad sleepers. It is worth considering that hormonal factors, such as the menstrual cycle, may contribute to gender differences in sleep quality (23, 24) and sleep disorders could affect the menstrual cycle (25). However, the menstrual cycle of the females was not controlled in this study and consequently, this is speculative. Our findings underscore the necessity to address gender disparities in the assessment and management of sleep quality in athletes. Nevertheless, it is essential to interpret our findings with caution due to the discrepancy in sample sizes between the two groups (males, *n* = 80; females, *n* = 32). It is important to note that this study was carried out during the training camps of the national teams, thus precluding selective sampling. This difference in sample sizes represents one of the main limitations of this study.

Regarding the specific components of the PSQI that exerted the most significant influence on overall poor sleep quality scores, all the variables measured exhibited significant differences between individuals categorized as good and bad sleepers. Nevertheless, sleep quality, sleep duration, and daytime dysfunction exhibited more pronounced distinctions between these two groups (see Table 3). This data holds potential value when considering potential group interventions aimed at enhancing sleep hygiene. Beyond group interventions, coaches and practitioners should also contemplate personalized interventions founded upon an analysis of individual scores.

Our results are in line with those previously published, demonstrating a high prevalence of sleep disorders in high-level athletes (3). As mentioned, this may compromise further adaptations and increase the risk of non-functional overtraining and injuries, as sleep is the most important contributor to recovery. The PSQI allows coaches and practitioners to perform individual sleep screening. In this regard, Walsh et al. (8) developed a flow diagram to help optimize and manage sleep for athletes, where sleep education plays a key role in the initial steps for addressing mild sleep problems and preventing future sleep disorders in athletes with good sleep behavior. This is important because our results showed poor sleep habits, even for the group of cyclists that scored <5 on the PSQI (Table 3). For example, the mean sleep onset latency was 45 min (Table 3), while this value should typically range between 10 and 20 min (26). Therefore, sleep education may benefit not only those cyclists with a certain level of sleep disruption but also prevent future declines in sleep quality among those cyclists with good sleep quality.

Based on our findings, different future research avenues emerge. Firstly, it is essential to conduct further investigations into gender differences in sleep quality, while considering hormonal influences as potential contributors. Moreover, longitudinal studies are needed to gain a better understanding of the relationship between the type of effort, sleep management strategies, and sleep quality over time. Additionally, it would be beneficial to explore interventions tailored to enhance sleep quality in cyclists, considering individual differences and sport-specific training demands. Lastly, it is worth noting that the incorporation of validated wearable sleep devices, known for their user-friendliness and minimal disruption to athletes during assessment, can offer a wealth of daily data (27). The data generated by these technologies could allow for more intricate and comprehensive sleep analysis. Indeed, several studies have assessed the benefits of fitness wearable devices, like wrist devices or smart watches for improving healthy habits (28, 29). Additionally, these devices could hold the potential to monitor and predict sleep dysfunctions in advance, thereby paving the way for more effective and proactive intervention strategies. Therefore, this area of research presents a promising avenue for future exploration. There are several limitations that should be considered when interpreting the results. First, the cross-sectional design only captures a snapshot of sleep quality, prohibiting any causal or longitudinal inferences. Second, the use of the Pittsburgh Sleep Quality Index (PSQI), while a validated tool, relies on self-reported data, which can be subject to recall bias and may not reflect objective measures of sleep patterns. Third, the study did not control for all potential confounding factors, such as stress levels, training load, and environmental conditions, which can affect sleep quality. Additionally, the hormonal influences, particularly among female cyclists, were not accounted for, which may have implications for the gender differences observed. Future research should consider these limitations and aim to include longitudinal designs, objective sleep measures, and a more diverse population to expand upon our findings.

In conclusion, the present study provides valuable insights into sleep quality among athletes in relation to different categories, gender, and the type of effort they engage in. Various facets of sleep quality were examined, encompassing different athlete categories, gender disparities, and training regimens. Notably, a significant proportion of elite cyclists displayed indicators of suboptimal sleep quality, suggesting potential areas for educational interventions, even among those initially classified as good sleepers. The use of the PSQI as a diagnostic tool holds promise for personalized interventions. These findings can serve as a basis for future research and the development of interventions aimed at improving sleep quality in cyclists, ultimately optimizing their performance and overall well-being.

## Data Availability

The raw data supporting the conclusions of this article will be made available by the authors, without undue reservation.
